# Historical Research: More Than Milk: The Origins of Human Milk Banking Social Relations

**DOI:** 10.1177/08903344221082674

**Published:** 2022-03-30

**Authors:** Tanya M. Cassidy

**Affiliations:** 1 School of Nursing, Psychotherapy, and Community Health (SNPCH), Dublin City University, Dublin, Ireland

**Keywords:** breastfeeding, donor milk, historical research, human milk, lactation, milk bank, prematurity

On September 5, 1909 the *Washington Post* ran a story entitled “Canned Mothers’ Milk,” (Figure 1). The newspaper discussion was short but nonetheless points to important details regarding the origins of what would be called—albeit much later (after World War Two)—human milk banking (HMB; Cassidy, 2009; Cassidy & Dykes, 2019). This is a history that considers the sociocultural importance of fame among physicians and scientists, but also fame associated with places, and how this celebrity relates to the control of lactation issues. It is also a story about links to the expansion of the first infant bovine food industry (most often cows’ milk, but not always), which is intimately tied to the expansion of scientific knowledge in the field of nutrition and reproduction. Finally, it is also a story about the concerns and considerations regarding the commercialization of infant foods, and, in particular, controlling—and often concealing—people who lactate.

**Figure 1.**
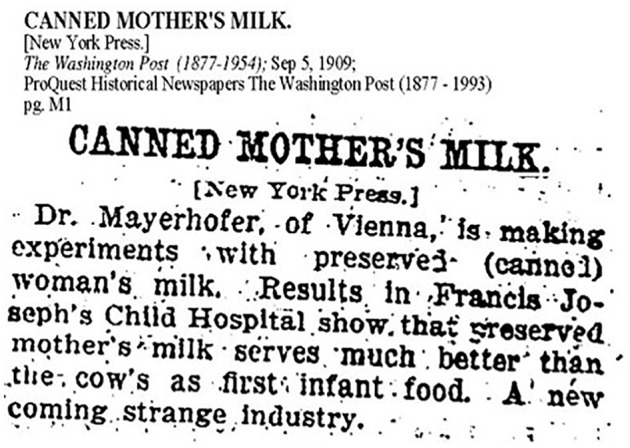
Canned Mother’s Milk. Note. From Washington Post (1909, p. M1).

Significantly, mothers’ own milk or breastfeeding is not mentioned in this article, and I would contend that in the Washington Post (1909) article this is not simply a feature of the commodification of human milk, which Golden (1996a, 1996b) argues eventually becomes understood as a gift as the 20th century progresses. A more recent sociocultural historical discussion by Rowold (2019) from the United Kingdom argued that this commodification is about the removal of the mother. However, as I discuss below, this removal occurs much earlier than is often argued and is tied to neonatal technology; it is much more about silencing the mother in these relations. By looking at the unspoken we can often trace important relations that have often gone unconsidered. The juxtaposition of product (milk) versus process (breastfeeding), to use the anthropologist Penny van Estrik’s (2015) formulation, is more about the recent anthropological discussions of the historically unspeakable (Dragojlovic & Samuels, 2021), which I am extending to consider other mothers’ milk. Social scientists, it has been argued, often discuss battles over thinking about “the social world” as primarily consisting in terms of substances or processes, in static “things” or in “dynamic, unfolding relations” (Emirbayer, 1997, p. 281). We will look at the dynamic social relations associated with these exchanges, including those underlying the processing of the milk itself. Ultimately, I wish to show that, by concentrating on the milk and forgetting the social relations, we run the risk of forgetting the people behind these exchanges, thereby neglecting the underlying links to breastfeeding, and falling into the conceptual trap of viewing human milk banking (HMB) on the same continuum as human milk substitutes.

## Expanding Medical and Scientific Fame, Technology, and Controlling Wet Nursing

One month after the *Washington Post* article appeared, on October 2, 1909, an unnamed “Special Correspondent” from Vienna appears in the *British Medical Journal* (*BMJ*), discussing these same issues and presenting rather more detail. The *BMJ* (1909) discussion begins by telling its readers that the work was presented before the Vienna Medical Society and was conducted by two doctors, Ernst Mayerhofer and Ernst Přibram (1909a, 1909b), who were members of Professor Escherich’s clinic.

Theodor Escherich is today remembered because of his ground-breaking work on what we now call *e-coli* (*Escherichia coli*; Hacker & Blum-Oehler, 2007). Widely recognized and respected, he was the only European pediatrician to be invited to speak to the International Congress of Arts and Sciences at the St. Louis World’s Fair on September 24, 1904, where he read the paper entitled *The Foundations and Aims of Modern Pediatrics* (Escherich, 1905; see also Shulman et al., 2007), which begins “Pediatrics, as far as it is connected with directions as to the care of the newborn and nurslings, belongs with midwifery to the oldest branches of medicine; but, in its scientific development, it is among the youngest” (Escherich, 1905, p. 55). The correspondent goes on to report that this “scientific development” did not occur until the beginning of the 19th century in France. According to the correspondent, following these early advances, the center of pediatric knowledge had shifted to Vienna, and the practice of pediatrics also had advanced across parts of Germany, a point made by a much more recent historian of milk studies.

In France, we see the lines between obstetrics and pediatrics blurred even further, particularly when we recognize that Etienne Stephane Tarnier (1828–1897) has been called the “architect of perinatology” (Dunn, 2002), which is the branch of obstetrics that concentrates on childbirth. Tarnier is remembered for his antiseptic studies, which greatly reduced maternal mortality, and also for his “new” forceps (Tarnier, 1877). The use of forceps is a practice with a long and interesting history, one cloaked, literally, in secrecy (Dunn, 1999). Tarnier also has been widely recognised as the inventor of the *couveuse* or incubator (Baker,1996, 2000), although his student, Pierre Budin (1907), said that others had introduced versions of this device earlier.

**Figure 2.**
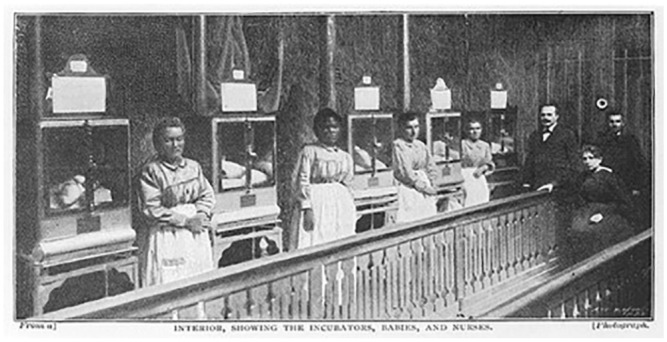
Lion Incubator in Use Attended by Dr. Lion, 1896. Note. From the Wellcome Collection. Attribution 4.0 International (CC BY 4.0) https://creativecommons.org/licenses/by/4.0/.

We have images from a number of these machines at the end of 19th century and into the beginning of the 20th century, but Figure 2, from the Wellcome Library, is especially interesting because, as the caption reads, the image depicts the “Interior, showing the incubators, babies, and nurses.”

The machines are argued to somehow “run themselves,” to the extent that the infants only need be fed and bathed, the role of their assigned nurses. It has also been pointed out that although the term “nurse” in English originally referred to “wet nurse,” it later took on its contemporary meaning to “take care of someone.” However, this linguistic change did not occur with the French or German terms *(la nourrice; die Amme)* where the original word for nurse still refers to wet nurse (Hill et al., 1987). In the case of the image in Figure 2, these nurses were in fact wet nurses.

Historians have often recognized that records regarding wet nurses are scant and often difficult to find (Apple, 1988; Fildes, 1986, 1988; Thorley, 2021). We should also consider anthropologists who use the term “allomaternal nursing” to refer to nursing from another mother, and who have argued that this practice is extremely common among all groups of peoples for whom we have records, both cross culturally and throughout history (Hewett & Winn, 2014). Contemporary discussions of cross nursing and peer to peer sharing discuss the secret nature of these exchanges (Shaw, 2004, 2007). We also know that there have been many narratives that demonized the women who nursed other women’s children and received some form of payment in return (Golden, 1996b).

Hill and colleagues (1987) also remind us that there were important distinctions between the practice of “rooming-out,” where an infant was sent to another’s home, often in the countryside (a practice which was more common in the 18th and early 19th centuries) versus “rooming in,” where the wet nurse moves into the home or, for the purposes of our discussion, the hospital. Many doctors talked about the difficulties of getting wet nurses to stay in the hospitals, and this increasingly seems to have been part of the larger problem of the medical control over wet nursing, although some hospitals kept complex directories, and would liaise between the hospital community and the wider community (Cassidy et al., 2019). It has been argued that rooming in resulted in reduced infant mortality rates, as wet nurses were monitored and under more direct medical supervision, as were the infants. Often, however, these wet nurses were from some of the poorest backgrounds and therefore ripe for potential exploitation. Rowold (2019) argued, albeit much later, that HMB in the United Kingdom resulted in the removal of the lactating body of mothers themselves. However, I would contend that this removal actually occurred much earlier in France, with the introduction of the gavage. Often forgotten is the fact that the gavage is also tied to both Tarnier and Budin, as the following quote demonstrates:

With excessive force-feeding, a very curious phenomenon occurs: the child rapidly increases in size and weight; but this increase is due to an edema which disappears with a more moderate diet, it can be explained by hypernutrition. But if, instead of reducing the quantity of the alimentary liquid, we maintained it, and especially if we increased it, we would not be long in observing indigestion, and the children would succumb with gastritis and enteritis: there is the greatest danger. To be successful, it is necessary that the milk is ingested in small quantities with each meal, except to multiply the meals. (Tarnier et al., 1888, p. 276–277; translated from the original using Google Translate)

Tarnier et al. (1888) go on to report that by using the gavage infants gained in size and weight; healthcare providers would also be able to control how much milk infants were receiving, as too much or too little could result in poorer outcomes.

**Figure 3.**
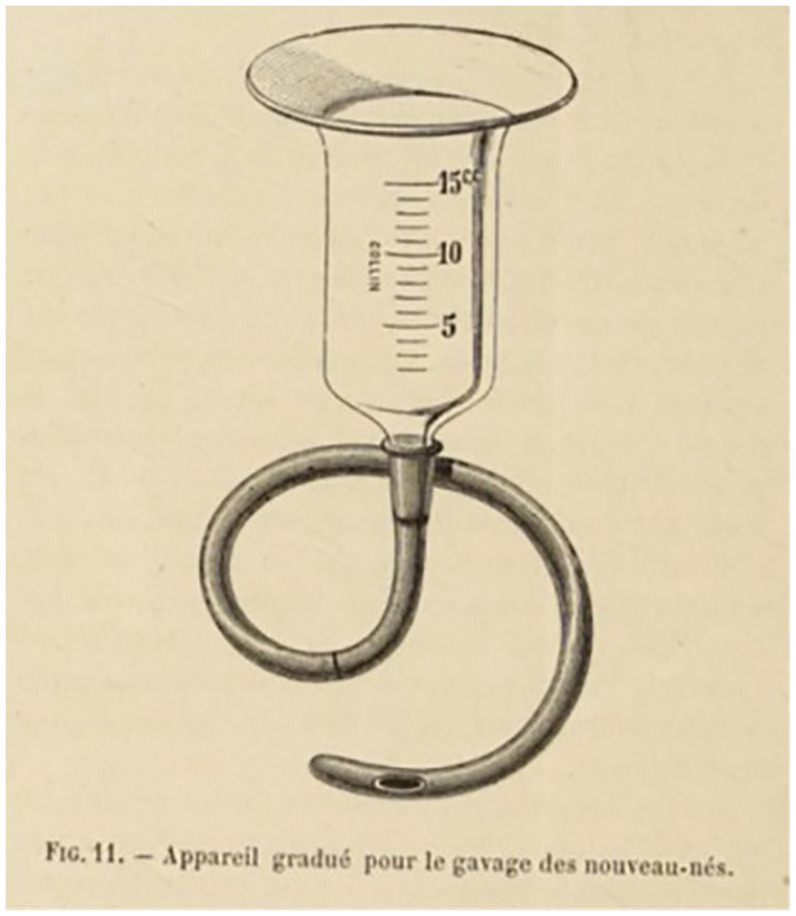
A Gavage. Note. From Tarnier et al., 1888, p. 276.

This gavage, unlike earlier versions, actually had measurement lines to aid the accuracy of intake (as the image in Figure 3 shows). This particular technological development is often forgotten, but it played a major role in controlling the “nursing” of these “weaklings,” as they were often called at this time (Greer, 2001).

Returning to Vienna 1909, another technology is mentioned whereby milk is drawn from a

Specially designed “breast-valve-pump” making it “possible to obtain all the milk contained in the gland by its action without the least discomfort to the woman . . . The advantage of this process lies in the fact that the milk can be drawn off whenever a wet nurse is present and can be kept until needed. As there are daily about 120 suckling women in Vienna lying-in hospitals, and each application of the pump easily yields three ounces, a quantity of about 120 pints could be had daily them (*BMJ*, 1909, p. 1005).

This brings us back to a discussion of wet nurses in Vienna. At the end of the 19th century there is a letter in the *BMJ* (1894, p. 1064) about a Wet Nurses strike in Vienna.

The grievances of Austrian wet nurses are numerous. Only the more radical among them insist on an eight hours labour day; many consider themselves entitled to keep the Sabbath as a day of rest from all work; and all of them *nem.con*. [without exception] ask for wages not less than 30 s a month and exemption from all kinds of work which lie outside the functions of wet nurses. But their main grievance, which is also the origin of the present agitation, is that they are systematically fleeced by professional agents who scour the country in search of wet nurses, receive a large premium on each from those who need her services—sometimes £5 or £6, besides a percentage from the woman herself (*BMJ*, 1894, p. 1064).

This letter lets us know that some people were making a lot of money from wet nursing, but not the wet nurses.

## Modification of Milk and the Science of Humanized Milk

Commercialization of infant feeding, however, was far more extensively related to milk from other animals, in particular cows, as we are told in the *Washington Post* (1909) article. We know that, in general, infants who received most of these non-human infant foods had poorer outcomes, leading to the extensive scientific studies to “humanize” these milks, which led to what some called “percentage feeding” or “formula” (Wolf, 2001). Wolf (2020) points out in her discussion about the origin of “formula” that infant feeding at the beginning of the 20th century dominated the thoughts of health care providers in many parts of the world. At the same time, we saw the development of the science of bacteriology, celebrating Louis Pasteur at the heart of its development. Although Pasteur’s work was originally designed to reduce spoilage in beer and wine, it was later applied by others to milk. Latour (1988) tells us that pasteurized milk from France was on display at the 1884 London International Exhibition. Wigner (1884) gave a lecture called *Pure Milk* at this exhibition, and declared that:

Human milk contains a larger proportion of sugar than cow’s milk, and less fat, caseine, albumen, and ash. It is from this that the formula generally adopted in the manufacture of artificial human milk obtained; cow’s milk is diluted with water, and then sugar added; by this means we obtain a liquid which assimilates somewhat closely in chemical composition to true human milk (p. 12).

Budin, and many other physicians, advocated the use of pasteurized—most often cows’—milk-based formulas for home use, the *Goutes de Lait* (Milk Drops), presumably assuming that mothers would breastfeed their own infants, at least for the earliest days, presenting a narrative reminiscent of the current World Health Organization’s arguments about exclusive breastfeeding for the first 6 months of life. We know that weaned infants had very high rates of mortality and morbidity and, for some, this form of purified milk contributed to these poor outcomes (Atkins, 1992, 2003).

**Figure 4.**
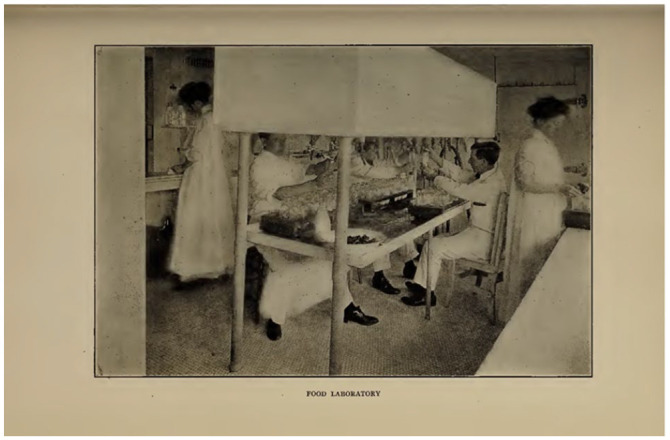
Boston’s Floating Hospital, 1910, p.43. Note. Thanks to the Boston Public Library.

As Wolf (2020) has discussed, the United States was particularly adept at the production of medically controlled formulae, but these same producers also had direct links with HMBs. Golden (1996a, 1996b) and Swanson (2014) have argued that HMBs began in North America at Boston’s Floating Hospital in 1910. The picture from their Annual Report (1910; Figure 4) shows the first season of the use of mothers’ own milk in the “food laboratory.”

**Figure 5.**
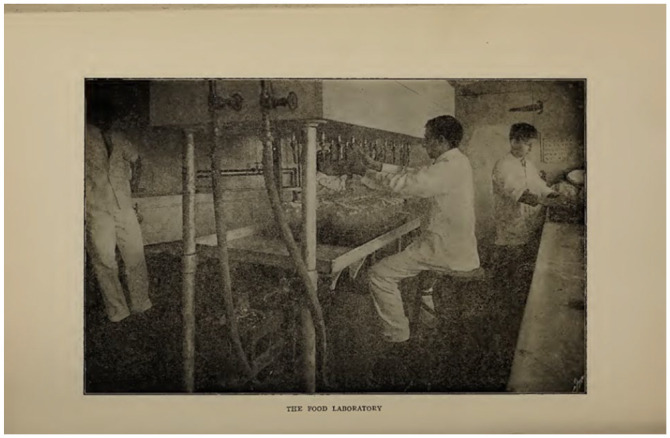
Boston Floating Hospital Annual Report 1907, p. 61. Note. Thanks to the Boston Public Library.

However, the “Food Laboratory” had been working since 1903, but had always been about cows’ milk, and was linked to Thomas Rotch and Harvard colleagues. Additionally, the laboratory was linked to Walker-Gordon farms, where Rotch had ties. The image from Boston Floating Hospital Annual Report 1907 (Figure 5), shows the “Food Laboratory” working with cows’ milk.

Note that there are no women present in this image. In fact, the first time a woman is linked to the human milk laboratory is in the 1910 image (Figure 4) when they reported bottling human milk as well. We are not told in the relevant Annual Reports how the milk—whether human or cow—was processed.

Discussion of the processing is relevant because then, as today, there are differences regarding the managing of milk. Returning to the *BMJ* (1909) discussion, we are told that they used the *Budde Method* for pasteurizing, and that the “milk is completely sterile,” which involved the addition of hydrogen peroxide along with heating the milk. This method was extremely well known and had been discussed in the *Lancet* in 1906 and was therefore widely recognized. More modern authors note that the term *Buddezing* was used across Europe (Hewlett, 1906).

## Why Social Relations Matter

Escherich died unexpectedly in 1911, shortly before the opening of the new children’s hospital in Vienna that he had helped design (Shulman et al., 2007). A year later, Mayerhofer and Přibram (1912) published another article detailing the successful use of preserved human milk with 100 infants. This was noted in the *Journal of the American Medical Association* and their German article was referenced (Ostheimer, 1912). However, if we read the original article we see that, on the first page, Mayerhofer and Přibram (1912) say that it was Escherich who first came up with this idea. They also say that they began these experiments in 1908, and mention some similar experiments, which were conducted by Knape (1911) from Magdeburg Hospital. There is another interesting part of this story to be told one day by someone with a closer German background, particularly since Magdeburg has held such an important place in the German history of HMB (see Steger & Kosenko, 2021).

When I first began looking into the history of HMB back in 2005, at the request of the global human milk banking community, I asked the question: Why did this service begin in Vienna? And why in 1909? As a cultural social psychologist interested in historical data, I framed my discussion abductively, using considerations from historical, economic, and feminist anthropology. A key anthropological economic concept is reciprocity (Sahlin, 1974), which Claude Lévi-Strauss (1969 [1949]) argued is a key feature of kinship, as well as central to gift exchanges. It draws on the work of Marcel Mauss (1987 [1950]), which I argue underlies human milk exchanges, particularly when we consider these issues from a historical feminist perspective.

Recently, we have been reminded that Mauss’ 1925 essay (2016 [1925]) originally began with a discussion about how social relations are forged by the ties of memory linking the living with the dead. In his now extremely famous essay, Mauss (2016 [1925]) argues that *“le don”* or “the gift” is not an expression of some rarefied and pure altruism but rather is impregnated with concepts of reciprocity in its very essence and countless applications. When milk is “gifted,” therefore, a relational chain of reciprocal obligations is activated (see Titmuss et al., 1997 for a guest chapter on milk banking and the original book from 1970). In economic terms, therefore, the “giving” of milk is part of a culture of exchange and expectation. As one donor from Vietnam remarked “giving is receiving.” This “gift theory” is based on a long tradition within Anthropology that continues to explore familiar forms of exchange, demonstrating complex ties to social relations and kinship, complicated by cultural considerations of gender and exchange (Strathern, 2020).

The medical control of wet nurses from the 18th Century onwards is a key consideration, but the use of gavage feeding from the mid-19th Century, and the potential feeding of infants without the mother, is also an important part of this history, demonstrating the complex longevity of the interaction between neonatal practice with technology, including the state-of-the-art breast pump, which was also mentioned in the original 1909 discussions. The breast pump is another important technology whose underlying social relations are key to the history of milk banking. Generally, milk technologies and the expression of human milk have proliferated. Equally important is to recognize that the science of preservation enables milk to be kept longer. The longer milk can be kept apart from the breasts and the lactating bodies that produced it, the greater the conceptual possibility of this milk being understood as “commodified.” Time (and with time, distance) emerges as an agent of de-personalization. However, this very depersonalization involves an understanding of social relations behind these behaviors. The commercialization of cows’ milk for preterm infants began in the mid 19th century (Schuman, 2003). However, there have always been commercial considerations and commercial anxieties associated with infant feeding—in particular with various allomaternal networks of exchange. There have always been fears that some mothers might want to make money from these developments, and these fears reinforce the medical control of commercial issues and the service itself.

Considering social relations not only between people, but also animals, is another key part of this consideration. Cohen and Ryan (2019) have presented a visual history, which includes connections between bovine relations and transportation, which can help to illustrate some of the direction I am suggesting here. Practices of inter-mammalian infant feeding have become framed as subversive, and utilized in constructing distinctions between humans and animals (Malcolm, 2021). Govindrajan (2021, 2018) has offered some important and interesting anthropological visions of interspecies social relations, arguing that we can better understand exchange relations by considering the “affective attachments” that “shape the ethics and politics of love,” by specifically looking at feminized labor experienced by cows, and specifically the cow’s “gift” of milk (Govindrajan, 2021, p. 195). We are only beginning to see some important and interesting anthropological visions of interspecies social relations, including animal/human discussions of cultures where cattle traditionally have a more sacred than an everyday social meaning. Cultural comparisons would also prove fruitful in the future to challenge the taken-for-granted meanings of interspecies relations.

HMB is an important part of hospital wet nursing, which was the first medical control of human milk; it first occurred in France, not Vienna. The use of gavage feeding lead to the removal of milk from the mother, and therefore the disembodiment of women in neonatal intensive care contexts. The gavage was also about measuring the milk and being able to control the infant feeding. In Vienna the organization becomes more linked to biological sciences, and to notions of fame and celebrity among these new medical scientists (e.g., Escherich). Twentieth century media technologies created new forms of “relationships” associated with celebrity which, in turn, influenced infant care. Considerations of medical control of infant feeding and the underlying potential for commercial considerations are decisive features of the emphasis on the milk, the commodity. However, this is not something accepted by the donors or by the HMB community, especially those who follow the World Health Organization’s award winning Brazilian Model tied to supporting breastfeeding (which traces its origins to a French system; Cassidy & Dykes, 2019; Langland, 2019). The French-Brazilian axis of influence deserves a separate future discussion. The notion of “gift” is about social relations underlying the exchange between not only people, past and present, but also animals and technologies. Within the world of HMB, we often recognize that women who provide milk remember the infants and their families whom they are helping to feed (Cassidy & Dykes, 2019). Therefore, social relations are involved in their breastfeeding relations in which these families play a major part. My overall argument is that we need to remember the people and the social relations underlying these milk exchanges. By doing this, we uncover more and better historical and cultural understandings.
